# Factors influencing sensitivity of a rapid influenza diagnostic test in a community‐based population of kindergarten through 12th‐grade students: Wisconsin 2015–2020

**DOI:** 10.1111/irv.13064

**Published:** 2022-10-31

**Authors:** Cristalyne Bell, Jennifer Birstler, Maureen D. Goss, Emily Temte, Shari Barlow, Guanhua Chen, Amra Uzicanin, Jonathan Temte

**Affiliations:** ^1^ Department of Family Medicine and Community Health University of Wisconsin‐Madison Madison Wisconsin USA; ^2^ Department of Biostatistics and Medical Informatics University of Wisconsin‐Madison Madison Wisconsin USA; ^3^ Division of Global Migration and Quarantine Centers for Disease Control and Prevention Atlanta Georgia USA

## Abstract

Rapid influenza diagnostic tests (RIDTs) have variable sensitivity. In a community‐based population of kindergarten through 12th‐grade (K‐12) students, we assessed factors that may influence RIDT performance using 2368 paired results from Sofia® influenza A + B fluorescent immunoassay and reverse transcription polymerase chain reaction (RT‐PCR). RIDT sensitivity and specificity were 76.1% (95% CI: 72.8–79.1) and 97.2% (96.2–97.9), respectively. Factors associated with sensitivity included runny nose (OR = 3.0, *p* < 0.001), nasal congestion (1.59, *p* = 0.045), days from symptom onset (per day; 0.75; *p* < 0.001), myalgia (0.61; *p* = 0.014), age (per 5 years; 0.55; *p* = 0.001), and detection of another virus (0.50; *p* = 0.043). Understanding these factors can aid in interpreting negative results.

## 1 BACKGROUND

Rapid influenza diagnostic tests (RIDTs) have long been used in clinical settings to identify patients infected with influenza. Studies have shown that point‐of‐care RIDTs reduce the use of antibiotics and improve clinical outcomes, but in many cases, test performance is unreliable.^1^ Although specificity is consistently over 90%, some studies report sensitivity rates as low as 17.8%.^2^ New advances in technology have improved overall sensitivity, but performance is still inconsistent and false negatives are fairly common.^3^, ^4^, ^5^, ^6^

The COVID‐19 pandemic resulted in a federal policy that allowed rapid antigen testing in homes, schools, and other community settings for the first time. This policy shift occurred, however, without formal evaluations of performance characteristics of specific over‐the‐counter tests in community settings. Many academic institutions conducted routine SARS‐CoV‐2 rapid antigen testing and experienced high specificity and varying sensitivity, similar to observations in clinical settings when testing for influenza.^2^

In the aftermath of the pandemic, we are likely to see the emergence of additional rapid testing technologies intended for school or community use. In addition to bolstering existing disease surveillance systems that are often reliant on clinical data, the near real‐time results in densely populated settings like kindergarten–12th‐grade (K‐12) schools may help contain outbreaks of influenza and other respiratory viruses. Accordingly, it is necessary to evaluate the performance characteristics of rapid tests in community settings. Such information can be essential to educating the public on how best to interpret results. Thus, we conducted a detailed analysis of the performance characteristics of Sofia® influenza A + B fluorescent immunoassay (FIA) to identify factors that could influence the sensitivity and specificity of RIDTs for K‐12 students in a community setting.

## 2 METHODS

### 2.1 Data collection

The ORegon CHild Absenteeism due to Respiratory Disease Study (ORCHARDS) is a prospective study of K‐12 student absenteeism and acute respiratory illness (ARI) in a community setting. ORCHARDS is ongoing and has been described in depth elsewhere.^7^, ^8^ The study is based in the Oregon School District, located in Southcentral Wisconsin. The presence of influenza within the school district is verified through home visits where research staff record demographic and symptom information and collect a nasal specimen and an oropharyngeal or a nasopharyngeal (NP/OP) specimen from eligible students. To participate, children must be experiencing at least two ARI symptoms that began within 7 days of a parent or guardian calling the study phone line, as well as a Jackson score of at least 2.^9^

Research staff perform a RIDT on the student's nasal specimen using the Quidel Sofia® influenza A + B FIA and notify the family of the result the same day as specimen collection, usually within 6 h of the home visit. The nasal swab is subsequently combined with the NP/OP swab in viral transport media and shipped via courier to the Wisconsin State Lab of Hygiene. The combined nasal and NP/OP specimens are tested for influenza A and B virus and Human RNase P using the CDC Human Influenza Virus Real‐time RT‐PCR Diagnostic Panel (Cat. # FluIVD03). Student specimens are also tested for non‐influenza respiratory viruses using a multiplexed PCR respiratory pathogen panel (RPP: Luminex NxTAG Respiratory Pathogen Panel).

For this analysis, we used data and specimens collected during six sequential influenza seasons—2014–2015 through 2019–2020. The ORCHARDS protocol was reviewed and approved by the University of Wisconsin Health Sciences Institutional Review Board.

### 2.2 Analysis

Our analysis was performed with R version 4.02 and used the same approach as our previously published analysis examining the performance of RIDT in a clinical setting.^10^ Binomial logistic regression models were used for adjusted associations. Adjusted models were fit for predicting improvements in sensitivity and specificity based on age, gender, number of days from symptom onset, presence and absence of individual signs and symptoms (chills, cough, sore throat, malaise, myalgia, runny nose, nasal congestion, and headache), presence of an influenza‐like illness (ILI), illness severity (mild, moderate, or severe), influenza vaccination status, co‐detection of other pathogens, and season (early, peak, or late). Specimens meeting inclusion criteria that had missing information were included in the overall sensitivity and specificity estimates, but they were removed from the adjusted model. We defined ILI as having a fever with cough and/or sore throat [23]. A quadratic term for age was not included because it did not significantly improve the model (LRT *p* = 0.46). Interaction terms between age and days from onset were also considered but ultimately excluded because of lack of significance (LRT *p* = 0.11). Some students had multiple specimens present in the dataset. Specimens, even from the same student, were assumed to be independent for testing and modeling.

## 3 RESULTS

Of the 2378 total student ARI episodes between January 5, 2015 and March 12, 2020, 2368 (99.6%) were included in this analysis. Nine specimens (0.38%) were excluded from the overall analysis because the child's symptoms began more than 7 days before the samples were collected, and one (0.04%) was excluded because an RIDT was not performed. For the adjusted model, 17 records were excluded because they were missing the severity of illness (*n* = 12; 0.50%) or were marked as missing the number of days from symptom onset because of an invalid test date (*n* = 5; 0.21%). Three (0.13%) specimens had inconclusive Sofia® test results. These were imputed as false positives in specificity analysis as all three had negative PCR results.

### 3.1 Participant characteristics

The average age of participants was 10.2 years with slightly more males than females represented (Table 1). More than half of the participants reported symptoms that met the definition of an ILI. Cough, malaise, and nasal congestion were reported in over 80% of participants, whereas runny nose and sore throat were reported in more than 70%. Myalgia was the least common symptom and was reported in 28% of participants. Slightly more than half of the children (52.4%) were vaccinated against influenza. Influenza was confirmed by RT‐PCR in 710 (30.0%) specimens (influenza A: *n* = 443; influenza B: *n* = 263; dual detection: *n* = 4).

**TABLE 1:** Demographics and distribution of sample characteristics for K‐12 students

Characteristic	Total, *n* (%)
Total specimens	2,368
Female	1018 (43.0)
Influenza‐like illness (ILI)	1392 (58.8)
Vaccinated against influenza	1241 (52.4)
Days from onset (mean ± SD)	1.93 ± 1.38
Age	
Mean ± SD	10.2 ± 3.47
Median [range]	9.95 [4–19]
Severity	
Mild	553 (23.4)
Moderate	1532 (64.7)
Severe	271 (11.4)
Season	
Early (July–November)	474 (20.0)
Peak (December–February)	1246 (52.6)
Late (March–June)	648 (27.4)
Symptoms	
Chills	1359 (57.4)
Cough	2042 (86.2)
Fever	1442 (60.9)
Headache	1491 (63.0)
Malaise	1994 (84.2)
Myalgia	662 (28.0)
Nasal congestion	1922 (81.2)
Runny nose	1678 (70.9)
Sore throat	1765 (74.5)
PCR results	
Influenza	710 (30.0)
A H1	191 (8.1)
A H3	251 (10.6)
A (other)	5 (0.2)
B	267 (11.3)

### 3.2 Rapid influenza diagnostic test performance

**FIGURE 1:**
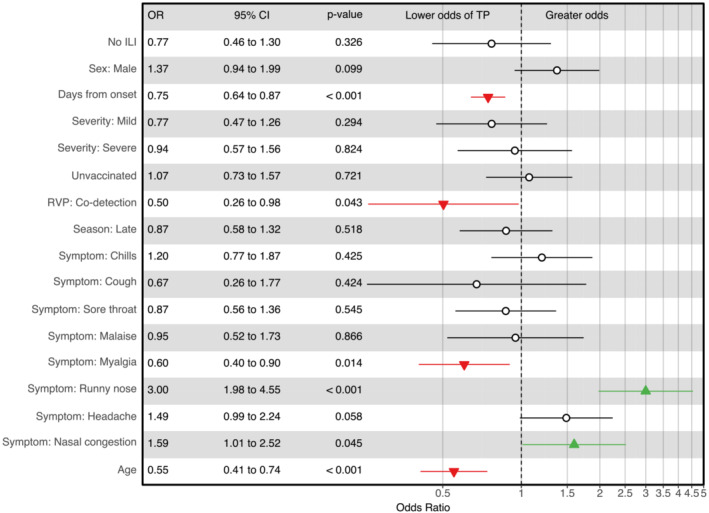
Odds ratios with 95% confidence intervals for factors associated with rapid influenza diagnostic test performance. RVP, respiratory viral panel; ILI, influenza‐like illness

**FIGURE 2:**
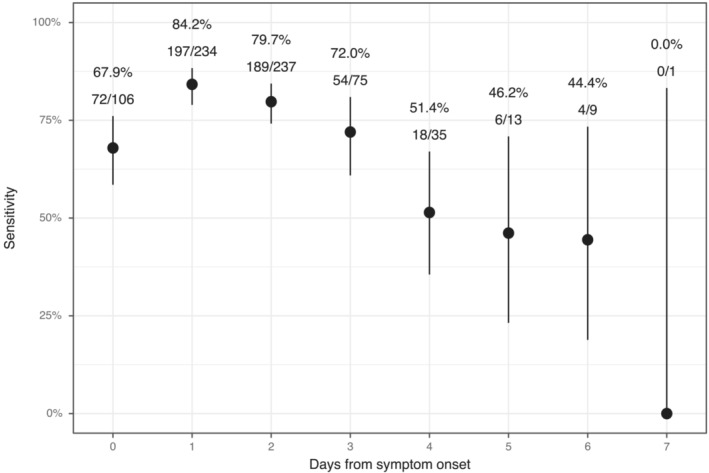
Sensitivity estimates with 95th percentile Agresti‐Coull confidence limits for Sofia® influenza A + B FIA based on days from symptom onset. The number of rapid influenza positive results are shown over the number of RT‐PCR positive results for each day.

Overall sensitivity and specificity of Sofia® were 76.1% (95% CI: 72.8–79.1) and 97.2% (96.2–97.9), respectively, with slightly higher sensitivity for influenza A than for influenza B (Table 2). Factors associated with sensitivity in the adjusted model were runny nose (OR = 3.0, *p* < 0.001), nasal congestion (1.59, *p* = 0.045), days from symptom onset (per day; 0.75; *p* < 0.001), myalgia (0.60; *p* = 0.014), age (per 5 years; 0.55; *p* < 0.001), and detection of another virus (0.50; *p* = 0.043) (Figure 1). Sensitivity was highest (84.2%) 1 day after symptoms began and dropped considerably by the fourth day (52.4%; Figure 2). Fever was excluded from the adjusted analysis because the variance inflation factor was 18.09, meaning it was highly collinear with other variables. None of the factors explored in the adjusted model were associated with specificity.

**TABLE 2:** Performance of Quidel Sofia® influenza A + B FIA

Measure	Overall	Influenza A	Influenza B
True positive	540	345	192
True negative	1611	1910	2057
False positive	47	15	41
False negative	170	98	78
Sensitivity (95% CI)	76.1 (72.8–79.1)	77.9 (73.8–81.5)	71.1 (65.4–76.2)
Specificity (95% CI)	97.2 (96.2–97.9)	99.2 (98.7–99.5)	98.0 (97.4–98.6)
Positive predictive value (95% CI)	92.0 (89.5–93.9)	95.8 (93.2–97.5)	82.4 (77.0–86.8)
Negative predictive value (95% CI)	90.5 (89.0–91.7)	95.1 (94.1–96.0)	96.3 (95.5–97.1)

## 4 DISCUSSION

Among a population of K‐12 students evaluated in a community‐based setting, Sofia® demonstrated high specificity and moderately high sensitivity. In an adjusted analysis, we found that older age, increased days from illness onset, the presence of additional viruses, and the presence of myalgia could result in lower sensitivity. Alternatively, the presence of runny nose and nasal congestion could improve sensitivity.

The effect of age on RIDT sensitivity is well documented, and our results demonstrate that this holds true even among children in a community setting.^3^ Previous studies have also noted the importance of the duration between when symptoms started and when the test was performed.^11^ The effect of individual symptoms on test sensitivity is not well understood, but our previous clinic‐based study also showed that the presence of a runny nose improves sensitivity.^10^ One explanation for this could be that having a runny nose or nasal congestion is correlated with a greater quantity of viral antigen within the sample collected. Absence of these symptoms may help guide the interpretation of negative results.

Our study benefited from a large sample recruited from a community setting over six influenza seasons, which is underrepresented in the literature where studies often rely on recruiting medically attended cases in healthcare facilities over fewer influenza seasons.^10^, ^11^, ^12^ Despite the strengths of the study design, our results should be considered in the context of at least two limitations. First, our analysis is based on the performance of one RIDT, and results may vary based on the type of test. Second, our study is limited to southcentral Wisconsin. Other studies have shown that sensitivity improves when prevalence is high, but local influenza seasonality is not well defined in the literature, so we defined it based on the general influenza season in the region.^12^

Our results suggest that routine use of RIDT in schools and other community congregate settings (e.g., select workplaces) could assist with early identification of increasing influenza activity and thereby help with timely implementation of targeted countermeasures. This in turn could help reduce virus transmission and related disruptions caused by widespread transmission, including increased student and staff absenteeism. Some real‐world experience of using RIDT to keep schools open already exists from the context of the still‐ongoing COVID‐19 pandemic. For example, in one study, continuous rapid testing after a known exposure performed as well as quarantine in preventing the spread of SARS‐CoV‐2.^13^ Future studies are needed to explore the feasibility of RIDT‐supported prevention programs to reduce the impact of influenza and other respiratory infections in other congregate community settings and in different populations.

## CONFLICTS OF INTEREST

Dr. Jonathan Temte has received past research funding from Quidel Corporation. Quidel provided in‐kind Sofia® tests for ORCHARDS. Quidel did not direct or exert any influence over study design, data collection and analysis, decision to publish, or preparation of the manuscript.

## AUTHOR CONTRIBUTION

**Cristalyne Bell:** Writing original draft; reviewing and editing; data curation. **Jennifer Birstler:** Formal analysis; methodology; visualization; writing original draft; reviewing and editing. **Maureen Goss:** Writing original draft; reviewing and editing; project administration; validation; data curation. **Emily Temte:** Data curation; project administration, validation; reviewing and editing. **Shari Barlow:** Project administration; supervision; reviewing and editing. **Guanhua Chen:** Formal analysis; reviewing and editing. **Amra Uzicanin:** Supervision; reviewing and editing. **Jonathan Temte:** Conceptualization; funding acquisition; reviewing and editing.

## ETHICS APPROVAL

All components of ORCHARDS were reviewed and approved by the Human Subjects Committees of the Education and Social/Behavioral Sciences IRB (initial approval on September 4, 2013; ID number: 2013–1268) and the University of Wisconsin Health Sciences‐IRB (initial approval on December 5, 2013, with additional approvals as the protocol expanded and modified; ID number: 2013–1357). The study is in full compliance with the Health Insurance Portability and Accountability Act of 1996 (HIPAA), Family Educational Rights and Privacy Act (FERPA), and all other federally mandated human subjects regulations. The US Office of Management and Budget approved all forms used in this study.

## PARTICIPANT CONSENT

Written informed consent was obtained from parents/guardians of minor students and from students aged ≥18 years.

### PEER REVIEW

The peer review history for this article is available at https://publons.com/publon/10.1111/irv.13064.

## Data Availability

Data for this study can be found at Harvard Dataverse: https://doi.org/10.7910/DVN/2NQ50C
